# Microbiology of the Gut of the Kola Nut Weevil, *Balanogastris kolae*

**DOI:** 10.1673/031.012.8401

**Published:** 2012-07-19

**Authors:** T. O. Femi-Ola, A. G. Babalola

**Affiliations:** Department of Microbiology, Ekiti State University, P.M.B. 5363, Ado-Ekiti, Nigeria

**Keywords:** bacteria, *Cola nitida*

## Abstract

Reports have shown that many insects have microbes in their gut system. Gut microbes are very important for insect vitality and much of their nutrition is derived from products of microbial metabolism. The habitat of *Balanogastris kolae* (Desbrocher des Loges) (Coleoptera: Curculionidae) suggests that they possess the ability to digest varieties of sugars particularly starch and protein materials present in the kola nut, *Cola nitida* Schott & Endlicher (Malvales: Malvaceae). The aim of this study was to characterize the gut bacterial communities of the kola weevil, *B. kolae*. To ascertain this, the gut bacterial community of a kola nut-feeding weevil, *B. kolae* was characterized using culture-dependent methods. The bacterial counts in the foregut, midgut and hindgut were 7.14 ± 0.11 × 10^6^cfu ml^-1^, 2.68 ± 0.13 × 10^7^ cfu ml^-1^ and 1.43 ± 0.20 × 10^6^ cfu ml^-1^ respectively. There were no significant differences in the total bacterial count of the foregut, midgut and hindgut. The bacterial species were identified to be *Fusobacterium nucleatum*, *Staphylococcus aureus*, *Bacillus subtilis*, *Corynebacterium fascians*, *Arthrobacter globiformis*, *Serratia marcescens*, *Bacillus brevis*, *Vibrio haemolyticus* and *Flavobacterium breve*. The majority of these isolates were demonstrated to have both proteolytic and amylolytic activities.

## Introduction

The weevils and other plantation pests are associated with a rich and complex community of microorganism in their guts and other body regions. This microbiota participates in many kinds of interactions ranging from pathogenesis to obligate mutualism (Dillon and Dillon, 2004). There has been a renewed interest in the understanding of insect gut, because they are potential source of enzymes (Zhang and Brune, 2004) and metabolites (Wilkinson, 2001). It has been reported that the manipulation of the microbial symbionts may be an effective strategy for controlling spread of pathogens that use pests as hosts (Dillon et al., 2005).

Weevils are the major pest associated with the spoilage of nuts and other crop plants. Among the weevil pests, the species found in Nigeria associated with kola nut are *Balanogastris kolae*, *Sophrorhinus insperatus*, *Sophrorhinus duverobyi* and *Sophrorhinus gbajensis*. The digestion of the kola nut by the kola-feeding weevil *Sophrorhinus insperatus* has been linked to the activities of enzymes present in the gut of the weevil. The dominant enzymes are the carbohydrases and proteases (Adedire, 1994). The enzymatic activities of the weevil have been reported to vary with the different segments of the gut; the foregut, midgut and hindgut. Enzymatic activities have been reported to be more prominent in the midgut (Adedire, 1994). However, there is a dearth of information on the microbial populations in the gut of kola nut feeding weevil and their possible role in the digestion of kola nut in the gut of the weevil. The knowledge of bacterial species composition will facilitate the studies of the functions of the gut microbiota.

The aim of this study was to characterize the gut bacterial communities of the kola weevil, *B. kolae* (Desbrocher des Loges) (Coleoptera: Curculionidae), using classical microbiological methods. This study also evaluates the production of some of the enzymes required for the digestion of kola nut in the gut of the kola feeding weevil.

## Materials and Methods

### Collection of materials

Kola nut weevils were collected from field-infested kola nuts, *Cola nitida* Schott and Endlicher (Malvales: Malvaceae) and were kept until emergence in a woven basket lined with banana leaves at insectary temperature of 28 ± 2° C and 80 ± 5% RH. The kola weevil samples were identified as *B. kolae*. There was no discrimination with respect to the sex of the insect used.

### Assessment of microflora in the gut of *B. kolae*

Approximately twenty weevil samples were selected for the isolation of gut bacteria. The weevils were anaesthetized in a refrigerator for 15 minutes and then surface sterilized by rinsing them in 10% v/v sodium hypochlorite and distilled water to kill external bacteria. Thereafter they were rinsed in two changes of sterile distilled water. They were aseptically dissected to remove the intestine. Each of the intestines was divided into three regions namely foregut, midgut and hindgut. The respective gut region was homogenized in 10 ml sterile Ringer's solution containing the following (g L^-1^): NaCl, 10.0; KCl, 0.42; CaCl_2_.2H_2_0, 0.48 and Na_2_HCO_3_, 0.2 (Femi-Ola and Aderibigbe, 2009). The gut sample was then crushed with a sterile glass rod to release the intestinal contents. Serial dilution of gut sample was carried out in sterile dilution blanks of distilled water and plated on nutrient agar (NA) plates in replicates and incubated at 37°C for 48h. Colonies on plates were counted to determine the total colony forming units of bacterial (cfu) counts. Pure cultures of the isolates were obtained by sub culturing serially onto sterile nutrient agar plates.

### Characterization and isolation of bacterial isolates

Pure cultures of the isolates were identified on the basis of their cultural, morphological and biochemical characteristics in accordance with the taxonomic scheme of Holt *et al*. (1994) and Barrow and Feltham (1993). The test performed include Gram-stain, spore-stain, motility test, Voges-Proskauer, oxidative/fermentative tests, gelatin hydrolysis, oxygen requirement, growth at different salt concentration and utilization of carbon source.

### Physiological studies of microorganisms in the gut of *B. kolae*

Bacterial isolates were screened for their amylase and protease activities.

Alpha amylase production was tested by inoculating the bacterial culture on starch agar (nutrient agar plus 1% soluble starch). Plates were incubated at 35° C for 3 days, after which they were flooded with iodine solution [0.3% 12 (w/v) in 3% KI (w/v)]. Amylase was indicated by a clear/brown zone surrounding the colony. Amylase activity was determined quantitatively by the dinitrosalicyclic acid (DNSA) method of Bernfield (1955), which measures the increase in the reducing power of the digests in the reaction between starch and the enzyme. One unit of alpha amylase activity was defined as the amount of enzyme required to produce one micromole of maltose from starch under the assay condition.

To determine of protease activity, bacterial isolates were inoculated onto casein agar plates and were incubated at 35° C for 24h. Protease production was indicated by a clear zone of casein hydrolysis. Protease activity was measured quantitatively by the method of Kunitz (1974). One unit of protease activity was defined as the amount of enzyme required to produce one micromole of tyrosine from casein under the assay condition.

### Statistical analysis

Analysis of variance of data obtained was carried out using the statistical package for Social Sciences (SPSS version 11.0).

## Results

The bacterial count in the different region of *B. kolae* is shown in Table 1. The total bacterial count in the foregut, midgut and hindgut were 7.14± 0.11 × 10^6^, 2.68± 0.13 × 10^7^ and 1.43 ± 0.20 × 10^6^ cfu ml^-1^ respectively. There was no significant difference in the total bacterial count of the foregut, midgut and hindgut (*p*≥ 0.05). The characterization of the bacterial isolates revealed that nine different species of bacteria were isolated from the gut of *B. kolae*. These bacteria were identified as *Staphylococcus aureus*, *Bacillus subtilis*, *Corynebacterium fascians*, *Fusobacterium nucleatum*, *Arthrobacter globiformis*, *Serratia marcescens*, *Bacillus brevis*, *Vibrio haemolyticus*, *Flavobacterium breves* and *Zymomonas mobilis* (Table 1). The rate of occurrence of the bacterial isolates is shown in Table 2. *B. subtilis*, *C. fascians* and *A. globiformis* had the highest occurrence rate of 66.6%.

Most of the bacterial isolates in this study were both proteolytic and amylolytic (Table 3), and *B. subtilis* showed the highest amylolytic and proteolytic activities. *S. aureus* and *F. breves* were only proteolytic and *F. nucleatum* did not hydrolyze either starch or casein.

**Table 1.**
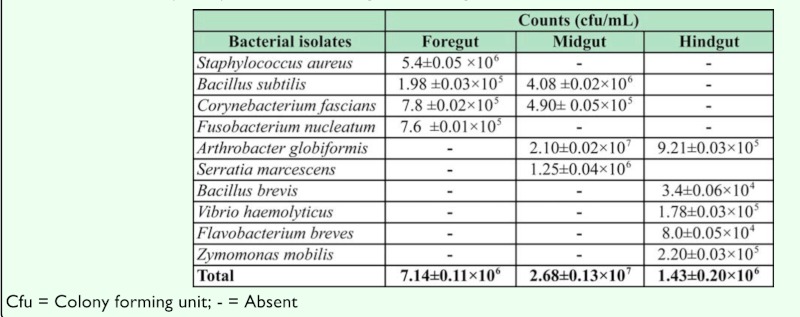
Total load (cfu/ml) of bacteria in the gut of *Balanogastris kolae*.

**Table 2.**
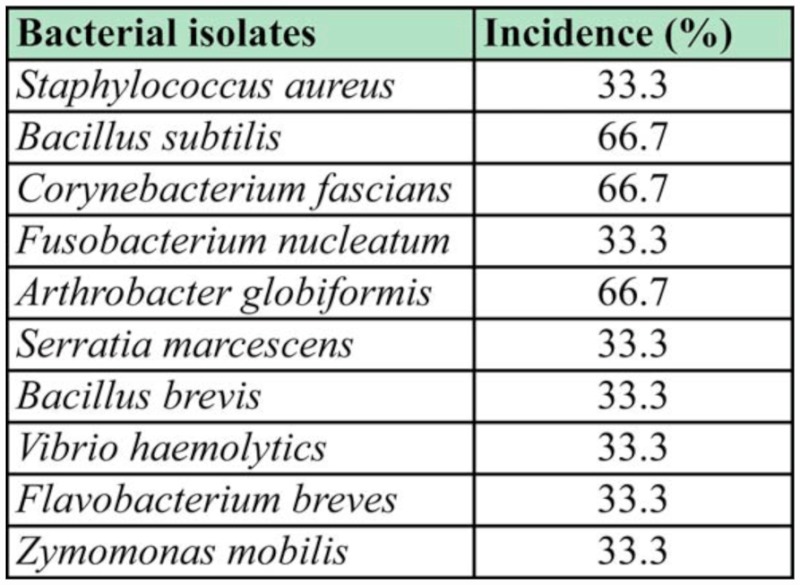
Rate of occurrence of bacterial isolates in the gut of *Balanogastris kolae*.

**Table 3.**
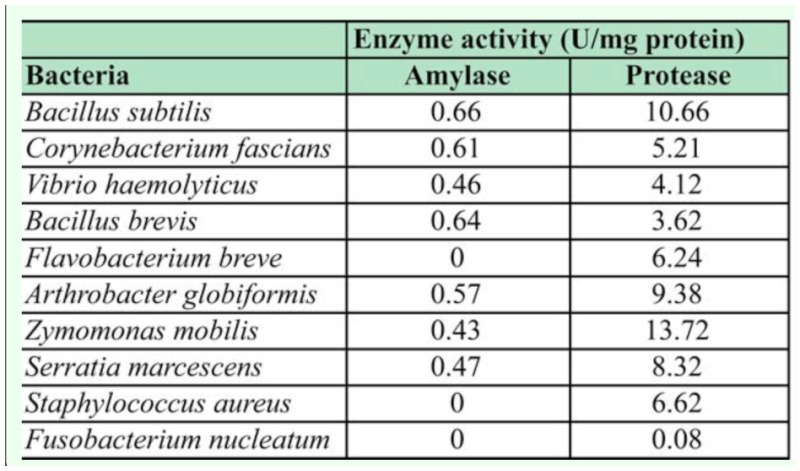
Enzymatic (amylase and protease) activities of bacteria isolated from the gut of *Balanogastris kolae* feeding weevil.

## Discussion

This study has shown that the kola weevil gut contained diverse bacterial species. *B. subtilis*, *A. globiformis* and *C. fascians* were isolated from two different regions of the gut, while the other bacterial species were isolated from a single region. This observation corroborates the report of Dillon and Dillon (2004), that bacteria are associated with a number of different insect species across all major orders of the insects. Eka (1971) had reported that the bulk of carbohydrate digestion takes place in the midgut of kola weevil. Carbohydrases and proteolytic activities have been reported by Adedire (1994) in the gut of the kola weevil, *Sophrohinus insperatus*, with the bulk of enzymatic activities in the midgut of the insect. In our study, most of the bacterial isolates were recovered from the midgut that is the site where most digestive activities (carbohydrate and protein utilization) were taking place compared to other sites of the gut. The high density of bacterial species observed in this region probably indicate the involvement of these organisms in the digestion of carbohydrate in the kola nut that forms the major diet of the weevil *B. kolae*.

Microorganisms in the gut of various insects have been reported to be involved in the degradative activities in the gut (Todaka et al., 2007; Bera-Maillet et al., 2000). In this study, most of the bacterial isolates were found to be both proteolytic and amylolytic. Some of these isolates may be relevant in relation to the diet of the insect as they produce enzymes that help to break down carbohydrate, protein and fats constituents of the weevil's diets. The organisms isolated are believed to be living in symbiotic relationship with the kola nut weevil that provides the food substrate for the micro flora in the different regions of the weevil gut. However, further research is needed to establish the roles played by the micro flora in the gut. Molecular studies of the gut will reveal the presence or absence of some non-culturable microorganisms that may be playing major roles in the in the digestion of kola in the gut of the kola nut feeding weevil.
